# GCN5L1-Mediated Lysine Acetylation Regulates Mitochondrial Bioenergetics and Redox Homeostasis in the Aged Heart

**DOI:** 10.3390/antiox15040481

**Published:** 2026-04-13

**Authors:** Jackson E. Stewart, Rahatul Islam, Ethan Meadows, Joshua P. Mogus, Murugesan Velayutham, Valery V. Khramtsov, Iain Scott, John M. Hollander, Dharendra Thapa

**Affiliations:** 1 Division of Exercise Physiology, School of Medicine, West Virginia University, Morgantown, WV 26506, USA; jes00050@mix.wvu.edu (J.E.S.); ri00005@mix.wvu.edu (R.I.); 2 Mitochondria, Metabolism & Bioenergetics Working Group, School of Medicine, West Virginia University, Morgantown, WV 26506, USA; emmeadows@mix.wvu.edu (E.M.); joshua.mogus@hsc.wvu.edu (J.P.M.); jhollander@hsc.wvu.edu (J.M.H.); 3 Department of Physiology, Pharmacology & Toxicology, School of Medicine, West Virginia University, Morgantown, WV 26506, USA; 4 In Vivo Multifunctional Magnetic Resonance Center, Department of Biochemistry and Molecular Medicine, School of Medicine, West Virginia University, Morgantown, WV 26506, USA; murugesan.velayutham@hsc.wvu.edu (M.V.); valery.khramtsov@hsc.wvu.edu (V.V.K.); 5 Division of Cardiology, Vascular Medicine Institute, University of Pittsburgh, Pittsburgh, PA 15260, USA; iain.scott@pitt.edu

**Keywords:** lysine acetylation, reactive oxygen species, mitochondrial bioenergetics, electron transport, aging

## Abstract

Precise control of mitochondrial electron transport is essential to maintain mitochondrial coupling and efficiency in ATP production. Furthermore, disruptions to ETC complex function can drive increased oxidant production, resulting in oxidative damage to the mitochondrion and bioenergetic inefficiency. This is highly relevant in the aging heart, as increased cardiac oxidative stress and mitochondrial dysfunction are hallmarks of age-related cardiovascular disease. Lysine acetylation has recently been characterized as a novel regulator of mitochondrial metabolic and bioenergetic function in the aging heart. In the present study, we investigated how lysine acetylation regulates oxidant production and redox milieu through mitochondrial acetyltransferase GCN5L1. Using a cardiac-specific GCN5L1 knockout mouse model, we observed that age-associated lipid peroxidation and semiquinone radicals were decreased with GCN5L1 KO. RNA sequencing analysis identified mitochondrial bioenergetic and respiratory pathways revolving around the respiratory chain to be enriched in the old KO group. Further, we showed the old KO group to exhibit reduced acetylation of ETC complex and antioxidant proteins, improved ETC complex and antioxidant protein activity. Overall, GCN5L1 regulates redox homeostasis in the aged heart by regulating mitochondrial ETC complex activity, oxidative stress, and mitochondrial bioenergetics. These findings identify GCN5L1 and acetylation as potential therapeutic targets in aging and age-related diseases.

## 1. Introduction

Cardiovascular disease (CVD) remains a significant cause of mortality among the aging population, with age playing a direct role in degradation of heart function by contributing to increased cardiac hypertrophy, myocardial fibrosis, hypertension, and myocardial infarction [1,2]. In 2019, the World Heart Report published by the World Heart Federation reported approximately 18.6 million global deaths attributed to CVD. With the increasing prevalence of obesity and diabetes, both significant comorbidities increasing the risk of CVD, the cost to treat patients suffering with CVD is expected to continue to increase worldwide [3]. Despite the prevalence of CVD, the precise mechanisms by which aging increases CVD likelihood and severity remain poorly understood [1].

Several cardiovascular pathologies have been associated with excess production of reactive oxygen species (ROS) [3]. ROS are collectively described as a group of oxygen-containing, highly reactive molecules, including both oxygen free radicals and non-radical oxygen derivatives. ROS constitute an important aspect of cellular function and are involved in several vital processes, including regulation of redox homeostasis, inter- and intra-cellular signaling cascades, regulation of vasodilation, and immune cell-mediated degradation of foreign pathogens [4]. While some ROS production is necessary, balance between ROS production and antioxidant capacity is paramount to healthy cell and tissue function. Overproduction of ROS can exceed the antioxidant capacity of the cell and damage macromolecules, namely proteins, lipids, and DNA. Damage to macromolecular structure can impair protein function, create mutations in DNA sequences, and inhibit lipid bilayer function in cell membranes, all of which contribute to cell dysfunction and, ultimately, cell death. Further, ROS overproduction has been shown to play a direct role in the impairment of cardiac contractile function as well [2]. Excessive ROS production and increased oxidative stress result in mitochondrial dysfunction and have a pathological impact on cardiac function, often becoming a significant contributor to cardiovascular disease progression [2,5].

Mitochondrial dysfunction has been implicated in the progression of aging and several aging-related conditions [6]. Mitochondrion is the largest source of cellular ROS production, thus placing mitochondrial dysfunction as a significant potential contributor to ROS-mediated cardiovascular dysfunction. Aging has been associated with increased mitochondrial ROS production [7]. Furthermore, increased damage to macromolecules including oxidatively impaired lipids, proteins, and damaged DNA are correlated with increased age. The mitochondrial respiratory chain is the primary source of ROS production within the mitochondria. The respiratory chain functions to maintain a proton gradient across the mitochondrial inner membrane by facilitating electron flow through the complexes in the chain and, ultimately, to molecular oxygen. However, under disease conditions, the respiratory chain becomes increasingly “leaky”, passing electrons to alternative locations largely at complexes 1 and 3 [8]. Instead of being passed to the next electron carrier or complex, electrons can be passed out of the chain to nearby molecular oxygen, generating a superoxide radical anion. This superoxide radical anion is converted to hydrogen peroxide by superoxide dismutase and self-dismutation. The increased ROS production leads to oxidative damage to mitochondria in a positive feedback loop that further worsens mitochondrial function and leads to more ROS production [9]. This loop becomes increasingly prevalent in the aged heart [10]. The current study aims to improve our understanding of the mechanisms surrounding the age-associated increase in mitochondrial ROS production in the heart and how this contributes to mitochondrial dysfunction and cardiovascular disease in aging.

Lysine acetylation is a post-translational modification of proteins that has been presented as a novel regulator of mitochondrial protein function in several cardiac pathologies including aging [11,12]. It has also been shown to regulate the activity and function of key antioxidant proteins such as manganese superoxide dismutase (SOD2) via acetylation at the K122 site, which has been shown to inhibit its catalytic function [13]. We have recently shown that the aged heart exhibits a pro-acetylation phenotype mediated by increases in mitochondrial acetyltransferase GCN5L1, which regulates cardiac mitochondrial metabolism [11]. However, the role of GCN5L1-mediated lysine acetylation in regulating electron transport chain function, ROS-production, and oxidative damage in the aged heart is not known. Therefore, using our novel GCN5L1 cardiac KO mouse model, we aim to assess the role of lysine acetylation in regulating mitochondrial respiratory function and redox milieu in the aged heart. We also aim to elucidate the mechanisms by which lysine acetylation impacts mitochondrial coupling and bioenergetics in the aged heart, thereby identifying potential points of therapeutic intervention for the improvement of treatment and prevention of cardiovascular disease in aging.

## 2. Materials and Methods

### 2.1. Animal Care and Use

Mice were housed at the West Virginia University animal facility under standard conditions with access to standard chow and water ad libitum. The mice were maintained on a constant 12 h light/dark cycle. Male and female GCN5L1 WT and KO mice, aged 4–5 months (control) or 24 months (aged), were sex-balanced in all experimental groups. The mice were euthanized via isoflurane/cervical dislocation, and heart tissues were excised for analysis. All experiments and procedures were conducted at West Virginia University and in compliance with the National Institutes of Health guidelines for animal care and use. All animal use protocols were approved by the West Virginia University Animal Care and Use Committee.

### 2.2. Transgenic Mouse Generation

Cardiac-specific tamoxifen-inducible GCN5L1 knockout mice were generated as previously reported [11,14,15]. Briefly, KO mice were generated via EUCOMM on a C57BL/6J background by crossing ⍺MHC-Cre mice (Jax B6.FVB(129)-Tg(Myh6-Cre/Esr1*)1JMK/J) with mice with LoxP sites flanking exon 3 of the BLOC1s1 gene encoding for the GCN5L1 protein (GCN5L1 FL). Cardiomyocyte-specific knockout was induced via single tamoxifen injection (40 mg/kg IP injection) at 2 months of age and confirmed with qPCR and immunoblot [11]. All WT animals received the same tamoxifen injection as KO to ensure observed differences are due to GCN5L1 cardiac KO and not off-target effects associated with tamoxifen or Cre recombination.

### 2.3. Protein and Mitochondrial Isolation

For heart protein lysate, heart tissues were minced and lysed in 1% CHAPS lysis buffer (1% CHAPS, 150 mM NaCl, 10 mM HEPES, pH 7.4) on ice for ~2 h. Heart homogenates were then centrifuged at 10,000× *g* and supernatants were collected for Western blotting, immunoprecipitation experiments, biochemical assays, and electron paramagnetic resonance (EPR) experiments. Mitochondrial subpopulations were isolated as previously described [16] with modifications [17,18,19,20]. Briefly, mitochondria were isolated from mouse heart tissue. Buffer 1 (100 KCl mmol/L, 50 MOPS mmol/L, 5 MgSO_4_·7 H_2_O mmol/L, 1 EGTA mmol/L, and 1 ATP mmol/L at pH 7.4) was used at a 1:10 weight/volume ratio to homogenize samples. The samples were then centrifuged at 700× *g* for 10 min. Following centrifugation, the supernatant was extracted and recentrifuged at 10,000× *g*. The precipitated pellet was washed in buffer 1 and centrifuged at 10,000× *g* twice. The precipitant from the 700× *g* spin was further processed by resuspending in a solution of KCl-MOPS-EGTA buffer (100 KCl mmol/L, 50 MOPS mmol/L, and 0.5 EGTA mmol/L at pH 7.4), and it was digested with 5 mg/mL of trypsin for 10 min. Following the 10 min incubation, a protease inhibitor cocktail (Thermo Scientific, Rockford, IL, USA) was added to the mixture to stop the digestion, and it was centrifuged at 700× *g* for 10 min. The supernatant was collected and centrifuged at 10,000× *g* for 10 min. Both sets of isolated mitochondria (from the initial supernatant and the digested pellet) were resuspended in 1% CHAPS lysis buffer and combined for a single total mitochondrial sample.

### 2.4. Electron Paramagnetic Resonance (EPR) Spectroscopy

Oxidizing potential of the mitochondrial environment was assessed using EPR spectroscopy with 1-hydroxy-3-carboxymethyl-2,2,5,5-tetramethyl-pyrrolidine (CMH). Briefly, isolated mitochondria resuspended in 1% CHAPS buffer were incubated with CMH for 30 min at 37 °C, followed by flash freezing in liquid nitrogen before storage at −80 °C until analysis. Reactive oxidants in the mitochondrial samples will react with CMH to form the EPR active CM^•^ radical [21,22]. The analysis was performed using a Bruker ELEXSYS E580 spectrometer (Bruker BioSciences, Billerica, MA, USA), operating at X-band with 100 kHz modulation frequency. The frozen samples were thawed to room temperature and immediately loaded (50 µL) into glass capillary tubes, sealed at one end using Critoseal clay, and then placed inside an EPR quartz tube (4 mm in outer diameter). The quartz tube was placed inside the resonator/cavity, and spectra were recorded at room temperature. The EPR instrument settings were similar to that of Velayutham et al. [22] as follows: microwave frequency, 9.854 GHz; sweep width, 100 G; microwave power, 23.77 mW; modulation amplitude, 1 G; modulation frequency, 100 kHz; conversion time, 29.3 ms; sweep time, 30 s; and number of scans, 1. The acquisition of EPR data was performed using the Bruker Xepr software (v1). The signal intensity was generated using the first peak (low-field) height of the spectrum.

EPR spectroscopic analysis was also used to measure the free radical and paramagnetic species in the mouse heart tissue. The excised heart tissues were flash-frozen in liquid nitrogen and stored at −80 °C until the EPR measurements were carried out. Each EPR sample was prepared by transferring the frozen heart tissue (160–270 mg) into a ceramic mortar prechilled with liquid nitrogen [23,24]. The tissue was then broken into small pieces in liquid nitrogen using a pestle. The tissue pieces were then loaded into a finger Dewar containing liquid nitrogen. The finger Dewar containing heart tissue samples in liquid nitrogen was positioned within the EPR spectrometer cavity. Low temperature (77 K) EPR spectra were recorded with a Bruker ELEXSYS E580 spectrometer operating at X-band frequency with 100 kHz modulation frequency and a HQ cavity as described previously [25,26]. The EPR data were collected using the Bruker Xepr program. All spectra were recorded with the following instrument parameters: microwave frequency = 9.467 GHz, modulation amplitude = 4.91 G (2 G for semiquinone radical), conversion time = 58.59 ms, scan time = 120 s, microwave power = 9.464 mW (0.95 mW for semiquinone radicals), and number of scans = 5. EPR spectra were plotted using the GraphPad Prism, version 10, software/program (GraphPad Software Inc., San Diego, CA, USA).

### 2.5. RNA Sequencing

For RNA-sequencing experiments, mRNA was isolated using a total RNA extraction kit (Qiagen, Germantown, MD, USA) according to the manufacturer’s protocols. Total high-throughput RNA sequencing, quality assessment, trimming, and alignment of sequences were performed using the RNA sequencing services available from Admera Health (South Plainfield, NJ, USA). Raw read quality was assessed using FastQC (v0.11.8) by MultiQC (v1.30). Adapters and poor-quality bases were trimmed using Trimomatic (v0.38), and reads were aligned using STAR Aligner (v2.7.1a). The resulting BAM files were marked mapping duplication using Picard tools (v2.20.4) and calculated to fragments per kilobase per million mapped reads (FPKM) using StringTie (v2.0.4). The identification of genomic features from the BAM files were completed using the Python tool HTseq-count (v0.11.2). Following HTseq, R studio environment (version 4.3.2—“Eye Holes”) was used for differential expression analysis and visualization. Any R libraries used in this study are depicted with brackets []. Counted reads from HTseq were transformed and normalized to log 2-like scale using variance stabilizing transformation (VST) [vst]. This was done to optimize homoscedasticity across the gene count set. VST-normalized counts were analyzed for differential expression using [DESeq2] to contrast each pairwise treatment condition comparison. Differentially expressed genes (DEGs) were considered statistically significant when *p* ≤ 0.05 for Wald tests following Benjamin–Hochberg adjustment. To identify DEGs with mitochondrial function, Ensembl gene IDs were matched to standard gene symbols using [org.Mm.eg.d] and then filtered for matched genes on the Broad Institute’s MitoCarta 3.0 curated mouse gene list (MitoCarta3.0: an inventory of mammalian mitochondrial proteins and pathways; Broad Institute). The VST-normalized counts for all significant mitochondrial DEGs were visualized in heat maps using [pheatmap v1.0.13].

### 2.6. Blue Native PAGE

To assess the quantity of assembled ETC complexes (I–V), blue native polyacrylamide gel electrophoresis (BN-PAGE) was performed as previously described [17,27] using equal amounts of protein. Briefly, isolated mitochondria were solubilized using 1% digitonin, and Coomassie blue G-250 was subsequently added. The samples were electrophoresed on 4–16% Native PAGE Bis-Tris gels (Invitrogen, Waltham, MA, USA). Gels were then fixed using a 40% methanol 10% acetic acid solution, followed by microwaving for 45 s at 1100 watts. Gels were decanted and destained with an 8% acetic acid solution, followed by microwaving for 45 s at 1100 watts an additional time. Gel was then placed on a rocker at room temperature until the desired background was obtained. To control destaining time and enable comparison between gels, each band of interest was expressed per the molecular mass marker 480 kDa band as previously described [27]. Each gel was then imaged, and densitometry was assessed using the ImageJ software (v1.54d, National Institutes of Health, Bethesda, MD, USA).

### 2.7. Western Blotting

For Western blot experiments, protein lysates were prepared using LDS sample buffer, separated using Blot-SDS 4–12% or 12% Bis-Tris gels, and subsequently transferred to nitrocellulose membranes (Life Technologies, Frederick, MD, USA). Protein expressions were analyzed using the following primary antibodies: rodent total OXPHOS antibody cocktail (Cat #ab110413) from Abcam, rabbit peroxiredoxin 5 (PRDX5) (Cat #17724-AP) from Proteintech (Rosemont, IL, USA), rabbit glutamate dehydrogenase (GDH) (Cat #14299-AP) from Proteintech, rabbit Thioredoxin 2 (Trx2) (Cat #13089-AP) from Proteintech, rabbit COXIV (Cat #3E11) from Cell Signaling, rabbit peroxiredoxin 3 (PRDX3) (Cat #10664-AP) from Proteintech, rabbit glutathione peroxidase 4 (GPX4) (Cat #52455) from Cell Signaling (Danvers, MA, USA), rabbit thioredoxin reductase 2 (TXNRD2) (Cat #16360-AP) from Proteintech, rabbit thioredoxin-interacting protein (TXNIP) (Cat #D5F3E) from Cell Signaling, rabbit catalase (Cat #D5N7V) from Cell Signaling, mouse SOD2 (Cat #66474-1-Ig) from Proteintech, and rabbit SOD2-K122 (Cat #ab214675) from Abcam (Waltham, MA, USA). Fluorescent anti-mouse secondary ((red, 680 nm) (Cat #92668070), (green, 800 nm) (Cat #92532210)) or anti-rabbit secondary antibodies ((red, 680 nm) (Cat #92668071), (green, 800 nm) (Cat #92632211)) from LiCor were used to detect expression levels. Protein densitometry was quantified using ImageJ software (National Institutes of Health, Bethesda, MD, USA).

### 2.8. Immunoprecipitation

For immunoprecipitation experiments, protein lysates were harvested in 1% CHAPS lysis buffer in the presence of deacetylase inhibitor (100 mM Trichostatin A and 5 mM Nicotinamide), and equal amounts of protein were incubated overnight at 4 °C with rabbit acetyl-lysine antibody (AcK; Cell Signaling). Immunocaptured proteins were isolated using Protein-G agarose beads (Cell Signaling Technology, Danvers, MA, USA), washed multiple times with CHAPS buffer, and then eluted in LDS sample buffer at 95 °C. Samples were separated on either 4–12% or 12% Bolt Bis-Tris gels and probed with appropriate antibodies. Protein densitometry was measured using ImageJ software (National Institutes of Health, Bethesda, MD, USA).

### 2.9. Electron Transport Chain (ETC) Complex Activity Assays

ETC complex activities (I, II, III, and IV) were measured in isolated mitochondria as previously described [17,28,29]. Briefly, isolated mitochondria from young and old WT and GCN5L1 cardiac KO animals were utilized to assess ETC complex activities individually. Complex 1 activity was assessed by spectrophotometric determination of NADH oxidation (absorbance at 340 nm) in the presence of decylubiquinone. Complex 2 activity was assessed by spectrophotometric determination of 2,6-dichlorophenolindophenol (DCPIP) oxidized (absorbance at 600 nm) in the presence of decylubiquinone. Activity of complexes 3 and 4 was assessed by spectrophotometric determination of reduced cytochrome C (absorbance at 550 nm) produced (complex III) or consumed (complex IV).

### 2.10. Mitochondrial Coupling Assay

The mitochondrial coupling assay was performed using a Seahorse XF Pro analyzer. Briefly, either an XF Pro plate or an XFe24 plate was hydrated one day prior to the experiment via addition of 175 uL (XF Pro) or 1 mL (XFe24) of calibrant solution to each well following by being covered with parafilm and placed in a CO_2_-free incubator overnight. On the day of the experiment, a fresh substrate solution of pyruvate + malate was diluted to 100 mM and 20 mM respectively. Additionally, four solutions of 40 mM ADP, 32 uM oligomycin, 40 uM FCCP, and 40 uM antimycin A + rotenone were prepared and loaded into ports A-D of the analyzer at 20, 22.5, 25, and 27.5 uL respectively to ensure 10× dilution in each well post-injection. The instrument was calibrated one hour prior to beginning the experiment. The instrument was programmed at 37 °C and set for two cycles of 30 s mix, 30 s standby, and 2 min data collection at the initial basal reading and following each injection. 2.5 uL of fresh isolated mitochondria were loaded into the wells at 1 ug/uL concentration followed by 20 uL of pyruvate + malate substrate. Wells were then loaded to 50 uL and the plate centrifuged at 1900× *g* for 20 min. Wells were then loaded to 180 uL total volume and the plate was placed in a CO_2_-free incubator for 10 min. After incubation the plate was placed in the analyzer and the experiment was performed. Data was generated using Wave Pro software (v10.3.0) and recorded as oxygen consumption rate over time. All data was exported to Prism Graphpad (v10.1.1) for analysis.

### 2.11. Biochemical Assays

Mitochondrial SOD and catalase activity were assessed in isolated mitochondria using commercially available kits according to the manufacturer’s protocols (Abcam #ab65354, Waltham, MA, USA and Cayman Chemical #707002, Ann Arbor, MI, USA respectively). Lipid Peroxidation was assessed in heart lysate using the Sigma-Aldrich MDA assay kit (Millipore Sigma #MAK085, St. Louis, MO, USA).

### 2.12. Statistical Analysis

Graphpad Prism software was used to perform all statistical analyses. Means ± SEM were calculated for all data sets. single-variable data sets were analyzed using 2-tailed Student’s *t*-tests. All multivariable data sets were analyzed using two-way ANOVA with Tukey’s post hoc multiple comparison tests or Šídák’s multiple comparison test for planned comparisons. *p* < 0.05 was considered significant. Total *n* of 2–7 was utilized depending on the experiment. Electronic laboratory notebook was not used.

## 3. Results

### 3.1. Markers of Oxidative Damage Are Reduced in the Aged GCN5L1 KO Mouse Heart Compared to the Aged WT Mouse Heart

**Figure 1:**
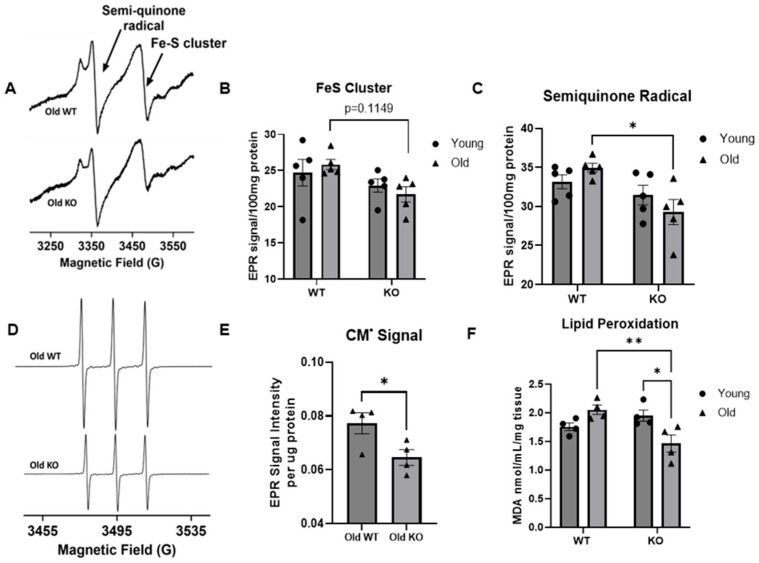
Measurements of oxidative potential in the mouse hearts. (**A**–**C**) EPR measurements of mitochondrial redox status in the hearts of young and old mice. Reduced oxidative stress in the aged GCN5L1 KO hearts. (**A**) Representative EPR spectra of heart tissue homogenates measured at 77 K. Semiquinone free radicals and Fe(III) from Fe-S centers are seen at *g* = 2.01 and *g* = 1.94, respectively. EPR signal amplitude was normalized against the weight of the heart expressed as arbitrary units per 0.1 g. (**B**) Normalized EPR signal amplitude of the Fe-S centers (*n* = 5 hearts). (**C**) Normalized EPR signal amplitude of the semiquinone radical (*n* = 5 hearts). Experiment details are given under the materials and methods section. (**D**) Representative EPR spectra of CM• radical measured at room temperature. (**E**) Summary data of CM• radical. Experiment details are given under the materials and methods section. (**F**) Lipid peroxidation (MDA) assay in heart lysate. Values are expressed as ±SE: *n* = 5–9; *p*-value significance was determined using two-way ANOVA with either Tukey’s post hoc multiple comparison tests or Šídák’s multiple comparison test for planned comparisons. Two-group comparison *p*-value significance was determined using Student’s *t*-test. * *p* ≤ 0.05, ** *p* ≤ 0.01.

Based on our previous findings showing how hyperacetylation of mitochondrial proteins in the aged heart was attenuated in our GCN5L1 cardiac KO aged animal and the acetylation-mediated disruptions present in mitochondrial substrate utilization and cardiac diastolic function in the aged heart [11], we hypothesized that these pathological changes would be underpinned by age-associated increases in oxidative damage in cardiac tissues. We thus performed EPR spectroscopy on young and old GCN5L1 WT and KO mouse heart tissue to examine semiquinone radical and iron–sulfur cluster radical formation. We observed a significant decrease in semiquinone radical content in our aged KO mouse hearts compared to aged WT, with a similar trend in iron-sulfur clusters (Figure 1A–C). Further, when we incubated isolated mitochondria with CMH, an EPR silent spin probe that becomes CM^•^ radical (EPR active) after reacting with oxidizing species in a sample, we observed a significant decrease in the signal obtained from our old GCN5L1 KO hearts compared to our old WT hearts (*p* < 0.05, Figure 1D,E), indicative of less oxidative stress in the absence of GCN5L1-mediated acetylation in the aged heart. We next assessed lipid peroxidation as a direct measure of oxidative damage to macromolecules and observed an increasing trend in the old WT heart compared to young WT. In agreement with the EPR data, we observed a significant decrease in lipid peroxidation in our old KO samples compared to old WT (*p* < 0.01, Figure 1F), implicating GCN5L1-mediated acetylation in the accumulation of oxidative damage to lipids in the aged heart. Taken together, these data suggest that GCN5L1-mediated hyperacetylation in the aged heart contributes to the overproduction of oxidative stress, and that GCN5L1 cardiac KO can ameliorate this increase.

### 3.2. The Aged GCN5L1 KO Mouse Heart Exhibits a Pro-OXPHOS Gene Expression Program

**Figure 2:**
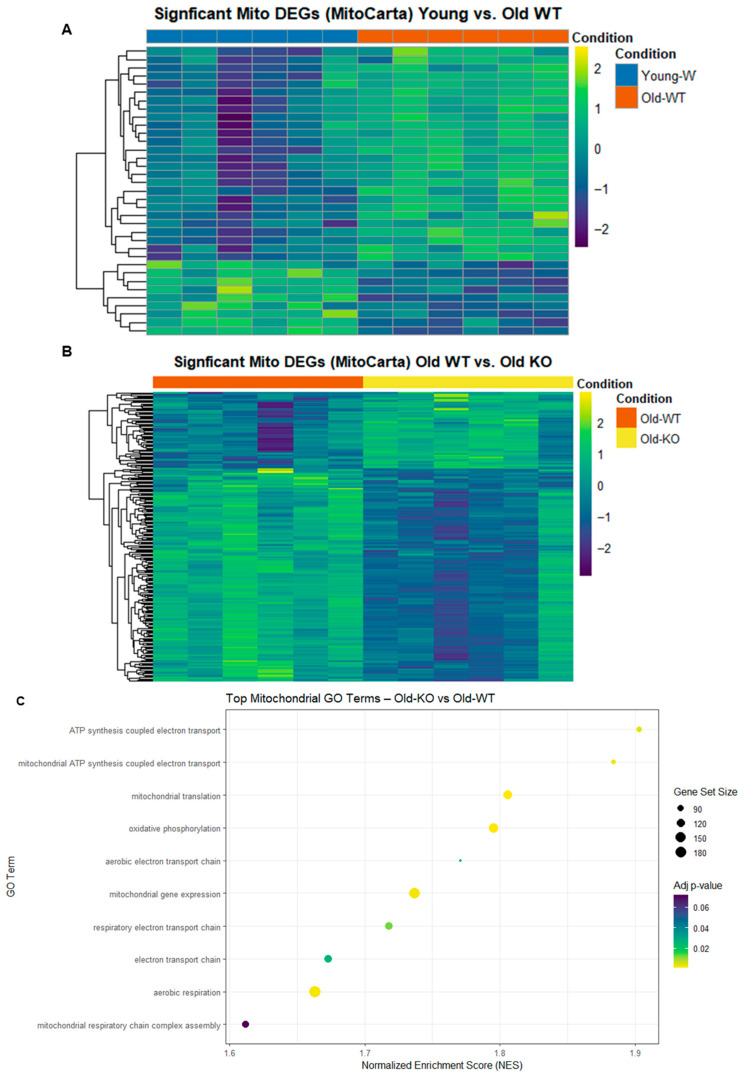
(**A**,**B**) RNA sequencing analysis of significant differentially expressed mitochondrial genes for young WT vs. old WT and for old WT vs. old KO. Color heat of genes indicates variance stabilizing transformed (VST) counts. Individual lanes indicate individual samples, color bar above heat maps indicate treatment condition groups (young WT = blue; old WT = orange; old KO = yellow). All genes included in heatmaps were significantly differentially expressed genes (Benjamin–Hochberg, *p* ≤ 0.05) included in the MitoCarta 3.0 mouse gene list. (**C**) Upregulated mitochondrial GO processes in the old KO heart compared to the old WT. Gene set size is denoted by the size of the dots in the plot, and *p*-value is denoted by color (all *p*-values ≤ 0.05).

**Figure 3:**
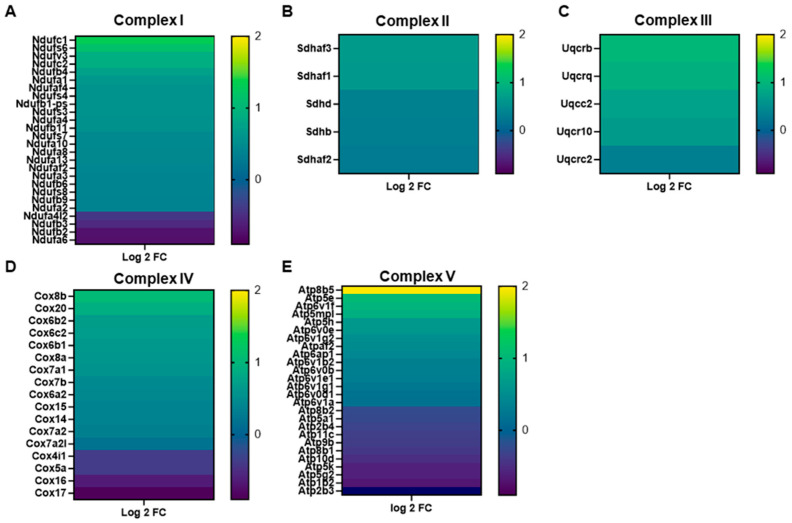
(**A**–**E**) RNA sequencing analysis for expression of ETC complex proteins from each mitochondrial respiratory complex in the old KO group compared to old WT. All significant DEGs for ETC complex proteins are displayed. *n* = 6; *p* < 0.05 was considered significant.

To identify potential molecular pathways that could contribute to the observed attenuation in mitochondrial oxidative damage in the old KO heart, we performed total RNA sequencing with gene ontology pathway analysis and calculated normalized enrichment scores for mitochondrial GO terms. We first observed a significant difference in mitochondrial differentially expressed genes (DEGs) between our old WT group and our young WT group (Figure 2A). When we compared the old KO to the old WT group, we observed a similar difference indicative of a significant change in mitochondrial gene expression resultant from GCN5L1 cardiac knockout (Figure 2B). Gene ontology analysis for mitochondrial pathways revealed a significant normalized enrichment score (NES) in several prominent mitochondrial respiratory pathways in our old KO group compared to old WT (Figure 2C), including oxidative phosphorylation, aerobic respiration, and ATP synthesis-coupled electron transport. Based on these data, we hypothesized that mitochondrial respiratory function could be improved in the old KO heart compared to old WT, and that this improvement could have further impacts on mitochondrial bioenergetic function and oxidant production in the aged heart. We next sought to determine the specific ETC-related transcripts that were impacted by genetic ablation of GCN5L1. We utilized our RNA sequencing data to compare expression of ETC complex proteins from all five complexes between the old WT and old KO groups (Figure 3A–E). We found that the old KO heart exhibits significantly increased expression of complex proteins from all five complexes, including increased expression of complex assembly factors that act as chaperones to aid in complex assembly and function. These data support our previous GO pathway analysis that genetic ablation of GCN5L1 results in a pro-OXPHOS gene expression program in the aged heart, potentially leading to improved complex function and ATP production.

### 3.3. The Aged Mouse Heart Exhibits Reduced Electron Transport Chain (ETC) Complex Assembly

**Figure 4:**
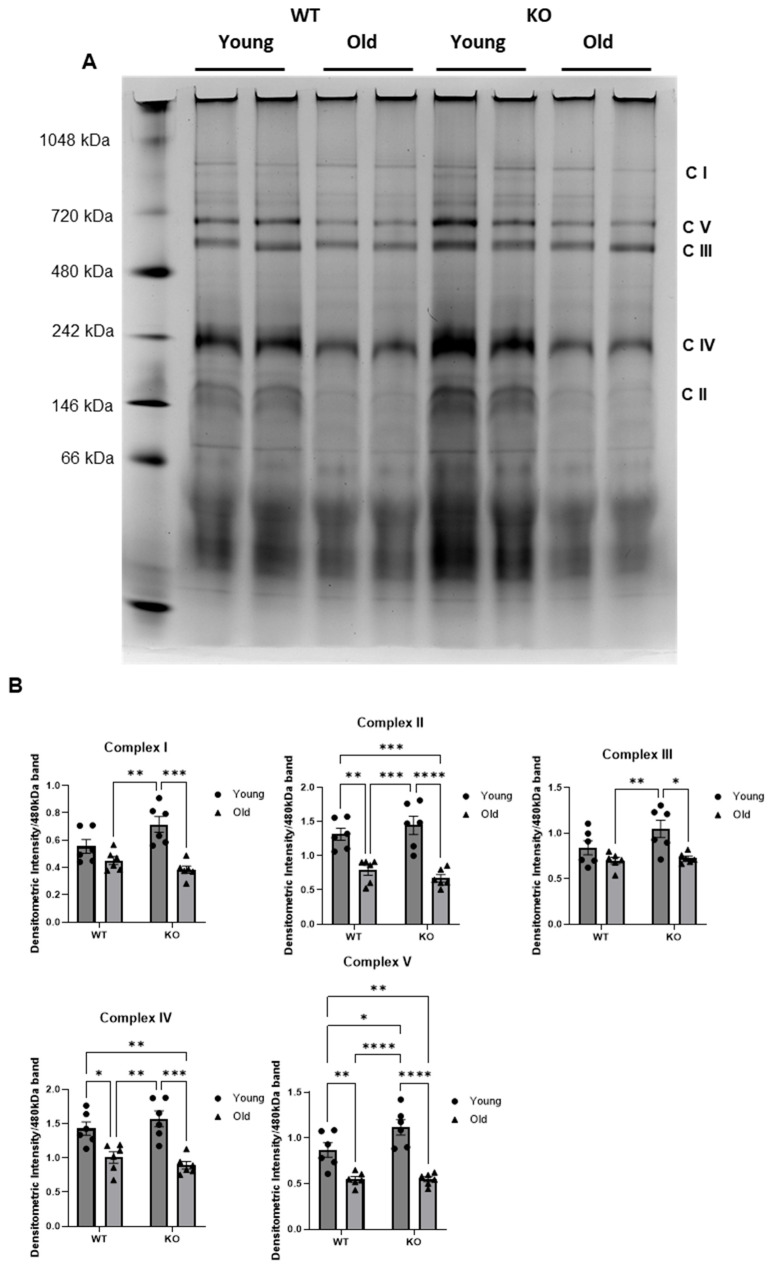
(**A**,**B**) Blue native PAGE analysis and quantification of mitochondrial electron transport chain complex assembly. Values are expressed as ±SE: *n* = 6; *p*-value significance was determined using two-way ANOVA with Tukey’s post hoc multiple comparison test. * *p* ≤ 0.05, ** *p* ≤ 0.01, *** *p* ≤ 0.001, **** *p* ≤ 0.0001.

To investigate the functional impacts of the pro-OXPHOS expression program we observed in our old KO hearts from our RNA sequencing data, we first assessed ETC complex assembly by performing Blue Native-PAGE using isolated mitochondria from young and old GCN5L1 WT and KO mice. We observed significant decreases in assembled ETC complexes II, IV, and V in the old WT heart compared to the young WT heart (*p* < 0.05 (CIV), *p* < 0.01 (CII and CV), (Figure 4A,B). Assembly of complexes I and III were reduced but not significant in the old WT heart compared to the young WT. We observed no significant differences in complex assembly between our old WT and old KO hearts, suggesting that GCN5L1-mediated lysine acetylation does not play a direct role in regulating total complex content in the aged heart. However, we did observe a significant increase in complex V in our young KO hearts compared to our young WT (*p* < 0.05, Figure 4B). These data suggest that ETC complex assembly is decreased in the aged heart, but GCN5L1-mediated lysine acetylation does not play a direct role in regulating complex content.

### 3.4. ETC Complex Proteins Are Hyperacetylated in the Aged Heart, Correlating with Suppressed Activity

**Figure 5:**
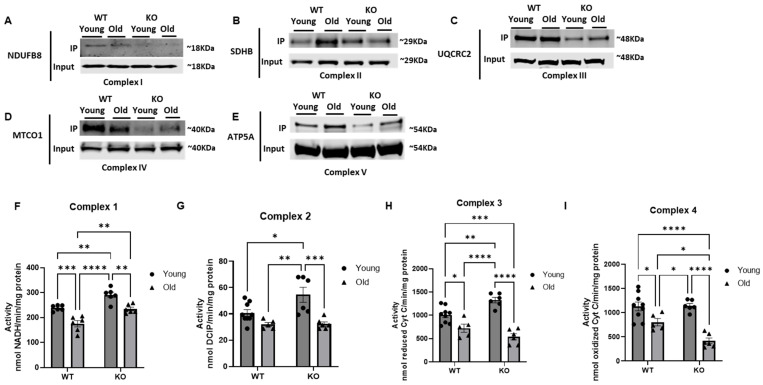
(**A**–**E**) Immunoprecipitation pulldown using a pan-acetyl-lysine antibody to assess acetylation levels of mitochondrial electron transport chain proteins from ETC complexes I–V. (**F**–**I**) ETC complex I–IV activity. Values are expressed as ±SE: *n* = 5–9; *p*-value significance was determined using 2-way ANOVA with Tukey’s post hoc multiple comparison test. * *p* ≤ 0.05, ** *p* ≤ 0.01, *** *p* ≤ 0.001, **** *p* ≤ 0.0001.

While we did not observe changes in ETC complex content between our old WT and old KO groups, we hypothesized that GCN5L1-mediated acetylation could still contribute to ETC complex dysfunction and oxidant production driving the oxidative markers we previously observed in the aged heart. We thus examined whether acetylation levels of ETC complex proteins were affected in our model. We performed acetyl-lysine immunoprecipitation with a pan-acetyl-lysine antibody, followed by Western blot for one representative protein from each ETC complex. We observed a robust decrease in the acetylation of all five complex proteins in our old KO hearts compared to our old WT (Figure 5A–E). In addition, we observed an increase in acetylation of SDHB (complex II) and ATP5A (complex V) in the old WT heart compared to the young WT (Figure 5B,E). Taken together, these findings suggest that GCN5L1-mediated acetylation could play a role in regulating ETC complex function independent of total complex content.

Based on this and our previous work demonstrating the correlation of increased acetylation with decreased activity of fatty acid oxidation proteins in the aged heart, we assessed whether GCN5L1-mediated hyperacetylation in ETC complex proteins in the aged heart could regulate complex activities. We thus performed individual maximal complex activity assays for ETC complexes I–IV and found an age-associated decrease in activity of complexes I, III, and IV (*p* < 0.001 (CI), *p* < 0.05 (CIII and IV) (Figure 5F,H,I), while complex II exhibited a non-significant decreasing trend (Figure 5G). When comparing our old GCN5L1 KO mouse hearts to the old WT, we observed a significant increase in complex I activity (*p* < 0.01, Figure 5F). Conversely, complex IV exhibits a significant decrease in activity in the aged KO heart compared to the aged WT (*p* < 0.05, Figure 5I). Taken together, these data suggest that GCN5L1-mediated acetylation plays a critical role in regulating ETC complex activity that is complex-dependent and that GCN5L1 KO could potentially decrease ROS production in the aged heart at the expense of maximal ATP production.

### 3.5. The Aged Mouse Heart Exhibits Significant Mitochondrial Respiratory Dysfunction That Is Not Present in the Aged GCN5L1 Cardiac KO Mouse Heart

**Figure 6:**
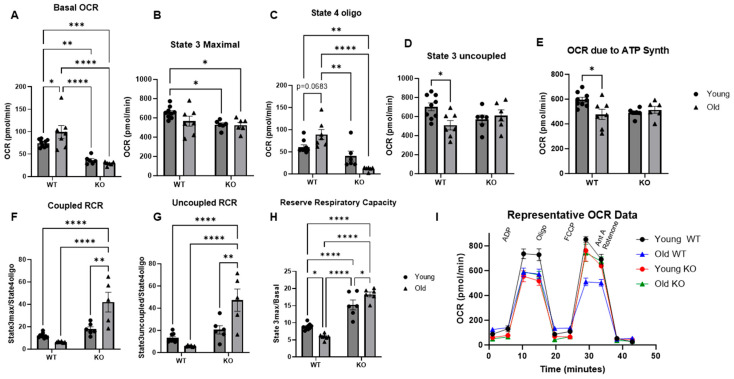
(**A**–**H**) Mitochondrial coupling assay assessing respiratory function. (**I**) Representative OCR tracings for each group. Values are expressed as ±SE: *n* = 7–9; *p*-value significance was determined using two-way ANOVA with Tukey’s post hoc multiple comparison test. * *p* ≤ 0.05, ** *p* ≤ 0.01, *** *p* ≤ 0.001, **** *p* ≤ 0.0001.

Based on our previous findings above, we wanted to further elucidate the role of acetylation in the age-related decline of mitochondrial respiratory function in the heart. We then performed a mitochondrial coupling assay using live isolated mitochondria and an Agilent Seahorse XF analyzer. We observed a significant increase in basal oxygen consumption rate (OCR) in our old WT samples compared to our young WT samples (*p* < 0.05, Figure 6A). Conversely, both our young KO and old KO groups exhibited significantly decreased basal oxygen consumption when compared to both the old WT and the young WT. We next stimulated State 3 maximal respiration via the addition of ADP to the samples. The old WT samples exhibited a decreasing trend when compared to the young WT, but the difference was not significant. Interestingly, both KO groups exhibited reduced maximal OCR when compared to the young WT (*p* < 0.05, Figure 6B). We then added oligomycin to inhibit ATP synthase and observed State 4 oligomycin-induced respiration, which is indicative of oxygen consumption that is largely due to electron leak from dysfunctional complexes. We observed a nearly significant increase in OCR in our old WT samples compared to our young WT, suggesting an increased electron leak in the aged cardiac mitochondria. Our young and old KO samples exhibited reduced OCR under State 4 conditions, indicative of exceedingly little electron leak (Figure 6C). Following this, FCCP was added as an uncoupler to observe State 3 uncoupled respiration. Our old WT samples showed significantly lower OCR compared to the young WT (*p* < 0.05, Figure 6D), while our young KO and old KO groups were not significantly different from the young WT. These data suggest that GCN5L1 cardiac KO alters mitochondrial respiratory function potentially in effort to decrease ROS production from aberrant electron leak. By facilitating a shift in mitochondrial respiratory function that prioritizes decreased electron leak at the expense of maximal respiratory capacity, the GCN5L1 KO mitochondrion could potentially be protected from the overproduction of ROS and subsequent damage thereby caused to the aging cardiomyocyte. Further, these findings are also supported by the increased ETC complex protein expression observed in the old KO heart from the RNA sequencing.

We then calculated several analytic parameters of mitochondrial respiratory function from the OCR values obtained over the course of the coupling assay. Firstly, OCR due to ATP synthesis is calculated as the difference between State 3 maximal and State 4 oligo OCR. We observed a significant decrease in OCR due to ATP synthesis in our old WT samples compared to our young WT samples (*p* < 0.05, Figure 6E). In our young KO and old KO groups, this significant decrease from the young WT was no longer present. Secondly, coupled and uncoupled respiratory control ratio (RCR) are general assessments of mitochondrial respiratory function and are calculated by taking the ratio of State 3 maximal or State 3 uncoupled OCR (respectively) to State 4 oligo OCR. We observed a decreasing trend in both parameters in our old WT samples compared to the young WT samples, suggesting decreased respiratory function under both coupled and uncoupled states. However, our young KO and particularly our old KO samples exhibited significantly increased RCR under both coupled and uncoupled states (Figure 6F,G). Lastly, reserve respiratory capacity (RRC) is indicative of an individual sample’s ability to respond to ATP demand and is calculated by taking the ratio of State 3 maximal OCR to basal OCR. We observed a significant decrease in RRC in our old WT samples compared to the young WT (*p* < 0.05, Figure 6H). However, both the young KO and old KO groups exhibited significantly increased RRC compared to the young WT and the old WT. This increase occurred despite the decreased State 3 maximal OCR shown in the KO groups largely due to a reduced basal OCR. Taken together, these data suggest that cardiac-specific GCN5L1 KO results in improved OCR due to ATP synthesis, improved respiratory control, and improved reserve capacity compared WT in the aged heart. Figure 6I provides a representation OCR trace for each group.

### 3.6. Antioxidant Protein Quantity and Expression Is Improved with GCN5L1 Cardiac KO

**Figure 7:**
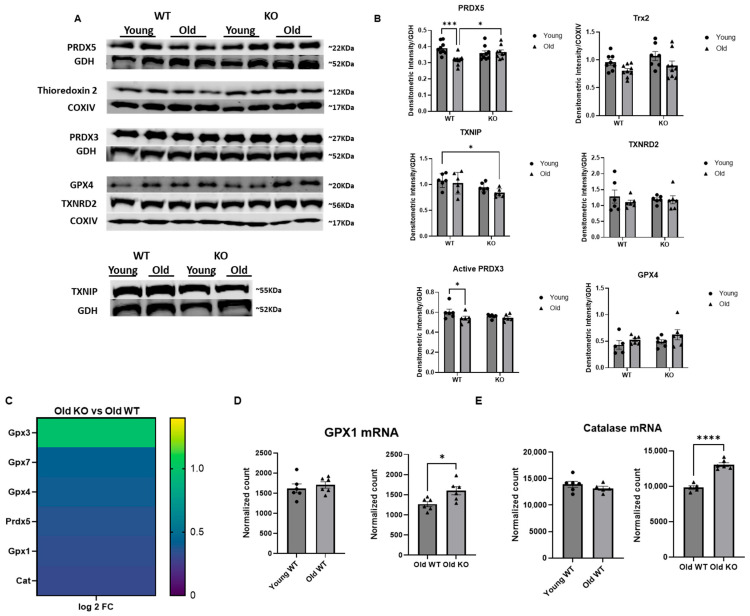
(**A**,**B**) Mouse heart lysate Western blot analysis for antioxidant proteins. (**C**) RNA sequencing analysis for expression of antioxidant proteins. All genes listed are significant DEGs for antioxidant proteins. (**D**,**E**) Normalized mRNA counts for *GPX1* and *Catalase*. Values are expressed as ±SE: *n* = 5–9; *p*-value significance was determined using two-way ANOVA with either Tukey’s post hoc multiple comparison tests or Šídák’s multiple comparison test for planned comparisons. *T*-test was used for single-comparison analyses. RNA sequencing heatmap: *n* = 6; mRNA counts: *n* = 5–6; *p* < 0.05 was considered significant. * *p* ≤ 0.05, *** *p* ≤ 0.001, **** *p* ≤ 0.0001.

Redox homeostasis is dependent upon balance between oxidant production and antioxidant defense. Based on our previous results revealing the role of acetylation in reducing electron leak in the aged GCN5L1 KO mouse heart compared to aged WT, we next sought to assess whether the balance between oxidants produced by the ETC and antioxidant proteins could be regulated in part by acetylation as well. As such, we performed Western blots with whole-heart lysate and probed for an array of antioxidant proteins. We observed a significant decrease in antioxidant protein peroxiredoxin 5 (PRDX5), as well as active mitochondrial PRDX3 (monomeric form) in our old WT animals compared to young WT (*p* < 0.001 and *p* < 0.05 respectively, Figure 7A,B). Conversely, we observed a significant increase in PRDX5 in our old KO samples compared to old WT (*p* < 0.05), suggesting a role for acetylation in degrading PRDX5 protein content with advanced age. In agreement with this finding, the significant decrease in PRDX3 observed in our old WT samples when compared to young WT is no longer significant when comparing old KO to young WT, indicative of improvements in active PRDX3 content in the age GCN5L1 KO mouse heart. Mitochondrial thioredoxin 2 (Trx2), albeit not statistically significant, decreased in the old WT compared to young WT and showed an increasing trend in the old KO compared to old WT. Mitochondrial thioredoxin reductase 2 (TXNRD2) and mitochondrial glutathione peroxidase 4 (GPX4) both exhibited no significant changes with age or GCN5L1 cardiac KO. We next measured the levels of thioredoxin-interacting protein (TXNIP) and observed no differences between our young and old WT groups. However, the old KO groups had significant reduction in TXNIP when compared to young WT animals (Figure 7A,B).

To corroborate our Western blot data, we created a heatmap of DEGs from our RNA sequencing data for antioxidant proteins comparing our old WT and old KO mouse heart samples. The RNA sequencing data revealed significant increases in several crucial antioxidant proteins in our old KO samples (Figure 7C), including *Cat*, *GPX1*, and *GPX4*, which is the predominant isoform of glutathione peroxidase in mitochondria. Additionally, raw mRNA sequence counts for *GPX1* and *Cat* both showed a similar increase in expression in the old KO group compared to old WT (Figure 7D,E). In support of the protein data, these data suggest that the old KO mouse heart exhibits improved antioxidant expression and protein quantity over the old WT, potentially leading to improved redox milieu with GCN5L1 cardiac KO.

### 3.7. Key Antioxidant Proteins, SOD2 and Catalase, Are Acetylated in the Aged Heart, Suppressing Their Activity

**Figure 8:**
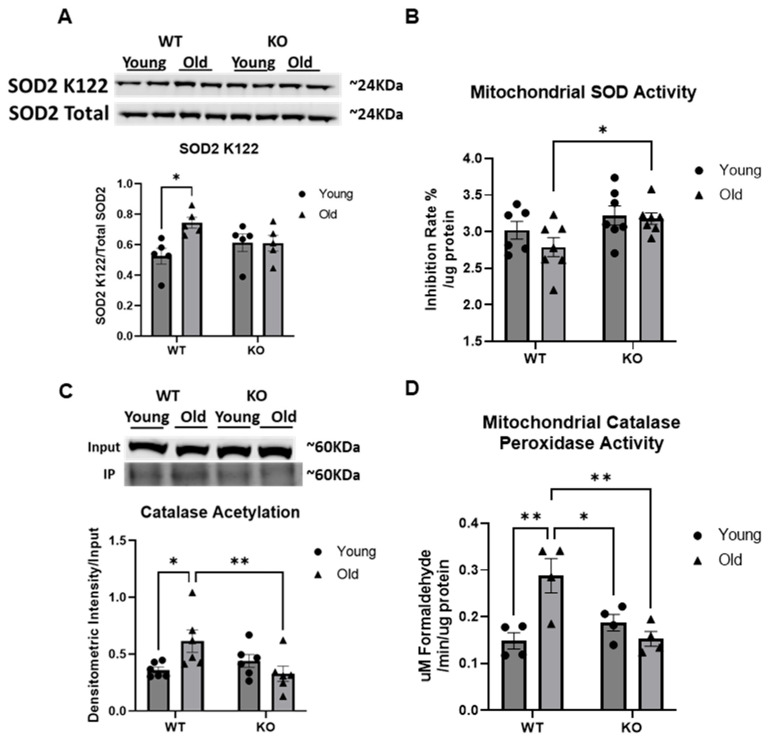
(**A**,**C**) Heart lysate WB Acyl-K IP for antioxidant proteins, SOD2 and catalase. (**B**,**D**) Mitochondrial SOD and catalase peroxidase activity assays. Values are expressed as ±SE: *n* = 4–7; *p*-value significance was determined using Student’s *t*-test for single-variable comparisons. For multivariate analyses, two-way ANOVA with either Tukey’s post hoc multiple comparison tests or Šídák’s multiple comparison test for planned comparisons. * *p* ≤ 0.05, ** *p* ≤ 0.01.

We next sought to assess the acetylation state of several key antioxidant proteins in the aged heart. SOD2 K122 was significantly increased in the old WT mouse heart when compared to the young WT mouse heart (*p* < 0.05, Figure 8A). This increase is no longer significant in the old KO mouse heart, demonstrating a role for GCN5L1 in regulating this age-associated hyperacetylation of SOD2 at the K122 site. When we assessed SOD2 activity, the old mouse heart exhibited a decreasing trend in comparison to the young mouse heart, and the old KO exhibited an increase in activity (Figure 8B). While the SOD activity data presented here does not show significant changes between groups, the trends in the data very nearly reach significance and reflect the changes we would expect to see in SOD activity when considering the acetylation data. Next, we assessed catalase acetylation in the same manner as above. The old WT heart exhibited a significant increase in catalase acetylation compared to young WT heart, which was significantly reduced in the old KO heart (*p* < 0.05 and *p* < 0.01 respectively, Figure 8C). When we assessed mitochondrial catalase peroxidase activity, we see a significant increase in the old WT heart compared to young WT heart, and the old KO heart catalase activity returns to young WT levels (*p* < 0.01, Figure 8D).

## 4. Discussion

Lysine acetylation has recently been shown to play a crucial role in regulating biochemical pathways and protein function in the mitochondrion. Mitochondrial deacetylase SIRT3 and acetyltransferase GCN5L1 have recently been characterized as novel regulators of diverse molecular pathways in several cardiac pathologies [12,15,30,31,32,33,34]. However, the role of mitochondrial lysine acetylation in the aging heart has yet to be thoroughly explored. Further, the contribution of GCN5L1-mediated lysine acetylation to the deterioration of mitochondrial redox milieu and bioenergetic function in the aged heart has not been examined. In the present study, we aimed to fill this knowledge gap utilizing our novel cardiac-specific GCN5L1 KO mouse model in young (4 months) and old (24 months) mice to investigate how lysine acetylation impacts mitochondrial redox homeostasis in aging. Based on our findings, we demonstrate for the first time that GCN5L1-meditated lysine acetylation regulates the acetylation status and activities of electron transport chain complex proteins, antioxidant proteins, mitochondrial coupling, and electron leakage in the aged heart. Furthermore, we show that aged GCN5L1 KO heart exhibits improved mitochondrial coupling, increased antioxidant protein content, and reduced markers of oxidative stress indicative of improved redox milieu.

Aging is a multifactorial pathology by which inherent biological imperfections lead to accumulation of damage to molecular and cellular structures, ultimately leading to cell senescence, cell death, and tissue dysfunction [35]. Although there is a myriad of subtypes of cellular damage, oxidative damage is critical in its contribution to mitochondrial dysfunction. Mitochondrial function is paramount to maintaining healthy cardiac function, as evidenced by mitochondria occupying one third of total cardiomyocyte volume [36]. Mitochondria also serve as the most prominent source of cellular ROS production via electron leakage from the ETC complexes. This electron leakage is exacerbated in the aging heart, leading to the accumulation of cardiomyocyte death and cardiac contractile dysfunction [37]. Therefore, identifying and studying the molecular mechanisms by which mitochondrial redox milieu is altered in aging hearts is critical for both basic and translational research to promote healthy cardiac aging.

One such mechanism reported to regulate mitochondrial function in several disease pathologies is lysine acetylation, which regulates cellular redox milieu and oxidative stress in several disease pathologies and tissues, including the heart [38,39,40,41,42,43,44]. However, many of these studies focus on the mitochondrial deacetylase Sirt3, which has been shown to improve redox milieu and promote pro-survival signaling cascades [38,45]. Therefore, there is a paucity of studies investigating the role of GCN5L1-mediated acetylation in regulating mitochondrial redox milieu, with none specifically addressing the aged heart. We have previously reported that the aged cardiac mitochondrion exhibits a pro-acetylation phenotype, and that this acetylation is correlated with dysregulated fuel substrate metabolism and cardiac diastolic dysfunction [11]. Fuel substrate metabolism is integrally linked with OXPHOS in the mitochondrion, the largest source of cellular ROS production. We first measured several markers of oxidative damage to assess the balance between oxidant production and antioxidant capacity in the aged heart. Our aged KO heart exhibits significantly reduced EPR signal after incubation with 1-CMH, which is indicative of a decrease in the oxidizing capacity of the cellular microenvironment. EPR signals from semiquinone radical and iron–sulfur clusters were also reduced in our old KO group compared to the old WT group. Furthermore, lipid peroxidation is decreased in the aged KO animals compared to WT, demonstrating a decrease in oxidative damage. ROS overproduction has been characterized as a primary driver of cardiac remodeling in heart failure with preserved ejection fraction (HFpEF), which is further characterized by cardiac diastolic dysfunction [46]. These data support our previous findings that the old KO heart exhibits improved diastolic function as evidenced by reduced E/A ratio when compared to old WT heart [11].

Based on our RNA sequencing data, we identified the mitochondrial respiratory chain as a primary target for our investigation into the role of GCN5L1 in regulating redox milieu in the aged heart. The measurement of ETC complex content using BN-PAGE showed a significant decrease in complex assembly across nearly all the complexes in the aged heart. Significant decreases in ETC complex assembly have been shown to be almost universally pathological [47,48]. Complex I deficiency is of particular significance, as it represents the most common defect in the respiratory chain and accounts for more than 30% of all mitochondrial diseases [49]. For example, specific mutations in the complex I protein NDUFS4 result in partial or complete loss of assembled complex 1. Assembly defects in complex I have also been linked to increased ROS production via altered activity at the flavin mononucleotide redox active site of the matrix arm [49]. Another group observed a significant correlation between decreased complex 1 assembly and enzymatic activity in MELAS cells, leading to reduced ATP production and increased NADH:NAD + ratio [50]. However, disease pathologies resulting from ETC complex deficiencies are not limited to complex 1. One case study of two human infant patients in 2011 discovered that a mutation in an assembly chaperone protein for ETC complex IV resulted in hypertrophic cardiomyopathy that further resulted in the death of both patients 8 and 10 days after birth, respectively [51]. Our data demonstrates significant reductions in complex content, likely contributing to ROS production and subsequent pro-remodeling signaling pathways driving the observed diastolic dysfunction present in old WT animals. ETC complex assembly is crucial for proper mitochondrial respiratory function, and any perturbations to complex assembly will be of high relevance in the heart due to its large mitochondrial count and high ATP demand for maintaining contractile function.

In our model, we show significant age-associated reductions in complex assembly in cardiac mitochondria. Because we did not see changes in complex assembly between our aged WT and aged KO samples, we conclude that acetylation does not play a significant role in regulating complex content. However, it is possible that the lack of significant differences in complex content between old WT and old KO groups could be due to increased mitochondrial turnover rates, leading to fewer damaged complexes and improved bioenergetic efficiency in the KO groups despite no change in total complex content. Future studies are warranted to investigate the role of GCN5L1-mediated lysine acetylation in regulating mitochondrial quality control mechanisms. Despite this limitation, we and other authors have previously reported that GCN5L1-mediated acetylation regulates the activities of several enzymes involved in fuel substrate metabolism [11,12,15,52]. We observed significant reductions in acetylation of proteins from all five respiratory complexes in our aged KO hearts compared to aged WT. Furthermore, significant decreases in complex I–IV were observed in the aged WT heart compared to the young WT heart, suggesting that the aged heart exhibited both reduced ETC complex assembly and activity. These reductions in activity could result in bioenergetic dysfunction and ATP depletion in the aged heart, potentially leading to cardiac dysfunction and pathological remodeling. In addition to these, dysfunction in complexes I and III could significantly increase ROS production and oxidative stress. Several studies have characterized the presence of mitochondrial respiratory dysfunction across tissues in the context of aging, and that this dysfunction drives oxidant production [53,54,55,56]. Interestingly, we observed a significant increase in complex I activity in our aged KO heart compared to the aged WT heart. This increase correlated with a decrease in acetylation of complex I protein NDUFB8, suggesting a role for GCN5L1-mediated acetylation in regulating complex I activity. Increased complex I activity in the aged KO heart suggests improved electron transport and potentially reduced ROS production, while decreased complex IV activity in the same group suggests reduced oxygen consumption and subsequent reduction in maintenance of the proton gradient and ATP production at complex V. ETC complex IV is largely regarded as one of the most significant rate-limiting steps in mitochondrial electron transport [57]. Reduced complex IV maximal activity may thus limit maximal electron flux through the respiratory chain and reduce maximal oxygen consumption. In support of this notion, we observed significantly reduced State 3 maximal oxygen consumption rate (OCR) in our old KO hearts compared to young WT in our mitochondrial coupling assay (Figure 6B), suggesting that while genetic ablation of GCN5L1 may improve ETC function in terms of minimizing electron leak, it may do so at the expense of maximal respiratory capacity through rate-limiting inhibition of ETC complex IV. These findings are in agreement with a report by Timkova et al. in 2016 examining the role of hyperhomocysteinemia on mitochondrial respiratory function [58]. Using a Langendorff perfusion model, Timkova et al. reported that homocysteine exposure significantly decreased the activities of ETC complexes II-IV with no apparent effect on complex I activity. Furthermore, they reported that this decline in ETC complex activity was not associated with any changes in oxidative damage, as indicated by unchanged protein thiol, carbonyl, or dityrosine levels. These findings support our own in the old GCN5L1 KO mouse heart, together suggesting that changes in individual complex maximal activities may not always be reflective of oxidant generation capacity. Complex I is one of the major sources of ROS production in the mitochondrial respiratory chain [56], and defects in its assembly and activity account for more than 30% of mitochondrial disorders, thus placing GCN5L1 as a significant potential regulator of mitochondrial function in aging through its impact on complex I.

Mitochondrial respiration is critical for proper mitochondrial function. We then performed a mitochondrial coupling assay to visualize electron transport and assess the activity of ETC complexes as a functional unit in live mitochondria. We show that the aged WT mouse heart mitochondrion exhibits significantly increased basal oxygen consumption rate (OCR), decreased oxygen consumption due to ATP synthesis, and increased electron leakage when compared to young WT mouse heart. Conversely, our aged KO animals show reduced basal OCR, improved OCR due to ATP synthesis, and decreased electron leak compared to the aged WT animals. These data demonstrate that GCN5L1 cardiac KO initiates a shift in mitochondrial respiratory function that prioritizes decreased electron leak at the expense of maximal respiratory capacity. Further, the aged KO also showed significantly increased RCR in both coupled and uncoupled states, as well as improved reserve respiratory capacity. Reduced basal oxygen consumption coupled with reduced respiratory control ratio (RCR) and reserve respiratory capacity have been shown to be characteristic of heart failure and dilated cardiomyopathy, as well as contributing mechanisms to cardiac ROS production and oxidative damage [8,59,60,61]. In our aged model, we demonstrate that aged WT mice exhibit significant cardiac mitochondrial dysfunction in the respiratory chain similar to heart failure pathology. Furthermore, we also demonstrate that by genetically ablating GCN5L1 and reducing hyperacetylation in the aged heart, we can attenuate the pathological perturbations to electron flow and OCR due to ATP production observed in the aged WT heart. One study demonstrated that metformin treatment improved cardiac and mitochondrial function in mice with heart failure (LAD ligation) via the upregulation of mitochondrial deacetylase SIRT3 and PGC1⍺ activity [60]. In our model, we demonstrate that age-associated hyperacetylation of the mitochondrial respiratory chain due to the upregulation of GCN5L1 results in derangements in mitochondrial ETC complex function, oxygen consumption, and electron leak.

Balance between ROS production and antioxidant defense is crucial for proper function. This is particularly relevant in the heart, as the high ATP demand and large mitochondrial volume increases its sensitivity to ROS production. Therefore, we sought to characterize how redox balance is impacted by GNC5L1 in the aged heart. Indeed, we observed age-associated decreases in antioxidant protein content in our old WT animals that was not present in our old KO animals. These findings suggest that GCN5L1 plays a critical role in regulating antioxidant levels in the aged heart. In addition, our RNA sequencing results also demonstrated significant increases in several antioxidant protein transcripts in our aged KO compared to aged WT mice, including catalase, PRDX5, and GPX4. However, the increase in mRNA level of GPX4 in Old KO hearts compared to old WT hearts did not result in increased GPX4 protein content, suggesting potential post-transcriptional or post-translational regulation at play. Further, it can also be caused by a high protein turnover/degradation of GPX4. These data suggest that GCN5L1 regulates an inter-organellar signaling interaction between the mitochondrion and nucleus, and that this process is dysregulated in the aged heart, leading to antioxidant protein deficiency coupled with increase ROS generation capacity from dysfunctional ETC complexes. Thioredoxin-interacting protein (TXNIP) is a pro-apoptotic protein that has been shown to inhibit thioredoxins, as well as translocate to the mitochondria under conditions of oxidative stress to initiate cytochrome C release and subsequent apoptotic cell death [62,63]. TXNIP deficiency has also been shown to inhibit type 1 and type 2 diabetes progression by inhibiting apoptosis of pancreatic beta cells [64]. When we assessed TXNIP content in our samples, we saw no significant difference between our young and old WT animals. However, our old KO heart samples had significantly less TXNIP when compared to young WT heart samples, suggesting reduced inhibition of the thioredoxin antioxidant system, as well as reduced apoptosis in the aged GCN5L1 KO heart. Taken together, these data suggest that the aged heart exhibits reduced antioxidant protein content that could potentially impair antioxidant response to ROS production via dysfunction in ETC complexes, and that this reduction in protein content is largely restored with GCN5L1 cardiac KO mice.

Antioxidant enzymes catalase and SOD2 were both hyperacetylated in the aged heart. The acetylation of SOD2 at the K122 site has been shown to be inhibitory of its antioxidant activity [65], and acetylation of catalase has been shown to increase its peroxidatic activity over its catalytic activity [66]. Reduced SOD activity and significantly increased catalase peroxidase activity present in aged WT animals was reversed in the aged KO animals, as we report increased SOD activity and significantly decreased catalase peroxidatic activity in the aged KO animals. Taken together, these data support our previous findings, showing that mitochondrial ETC complex dysfunction, increased oxidant generation, and reduced antioxidant capacity in the aged heart is integrally linked to aberrant hyperacetylation mediated by GCN5L1. However, while it is well known that acetylation can regulate protein enzymatic activity, elucidating the mechanisms surrounding GCN5L1-mediated regulation of antioxidant protein expression warrants further study.

While the study establishes the acetylation mediated role of GCN5L1 in regulating redox homeostasis in the aged heart, it should be noted that acetylation-independent roles of GCN5L1 have been reported as well. GCN5L1 interacts with aTAT1 and RanBP2 to regulate a-tubulin acetylation and lysosome trafficking (Wu et al. [67]). Furthermore, protein-interactome analysis have reported GCN5L1 to be associated with mitochondrial crista complex MIC13 and protease YME1L to regulate cristae morphology (Xu et al. 2025 [68]). Therefore, one of the limitations of this study is the inability to distinguish the impact of acetylation-independent mechanisms in our reported findings. Future studies focused on understanding this effect are warranted. Both male and female mice were balanced in each experimental group, and preliminary results showed very little difference between sexes within groups. Therefore, the influence of acetylation in regulating sex-based differences in cardiac mitochondrial metabolism was not addressed in this study.

## 5. Conclusions

**Figure 9:**
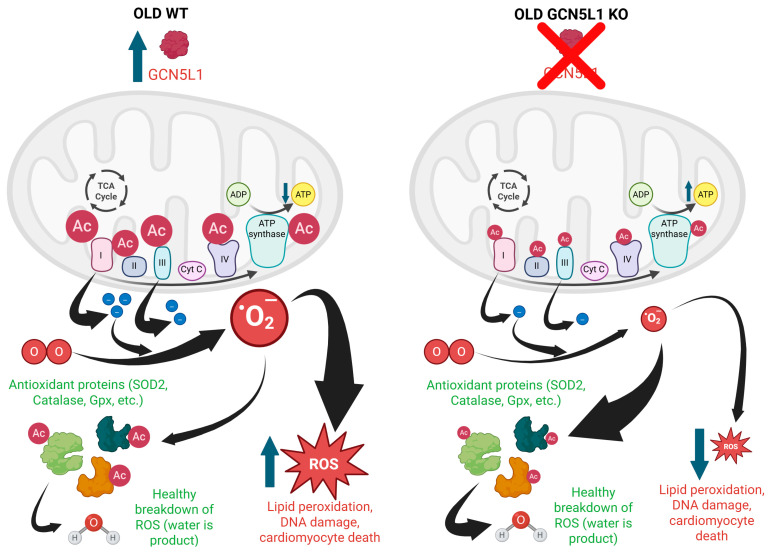
Graphical summary demonstrating the role of GCN5L1-mediated lysine acetylation in regulating mitochondrial respiratory function and redox environment in the aged heart (Created in BioRender. Thapa, D. (2026) https://BioRender.com/bsf59do) accessed on 1 December 2025.

Taken together, our findings characterize GCN5L1 as a novel regulator of mitochondrial redox milieu in the aged heart. Utilizing a novel GCN5L1 cardiac knockout model, we show acetylation-mediated regulation of mitochondrial OXPHOS function and antioxidant defense in the aged heart. Future studies will be focused on characterizing the specific role and mechanism that GCN5L1 has in regulating the deterioration of mitochondrial structure and function in the aged heart. These findings are presented in a graphical summary in Figure 9.

## Data Availability

The original contributions presented in this study are included in the article. Further inquiries can be directed to the corresponding author. The RNA sequencing data presented in this study is openly available in the NCBI Sequence Read Archive at https://dataview.ncbi.nlm.nih.gov/object/PRJNA1403444?reviewer=tosg8paf2k0e2h56hs2h9lja6i (BioProject Accession: PRJNA1403444) (accessed on 1 April 2026).

## Abbreviations

GCN5L1: general control of amino acid synthesis 5-like 1, ATP: adenosine triphosphate, ETC: electron transport chain, KO: knockout, CVD: cardiovascular disease, ROS: reactive oxygen species, SOD2: manganese superoxide dismutase, EUCOMM: European conditional mouse mutagenesis, qPCR: quantitative polymerase chain reaction, TEM: transmission electron microscopy, EPR: electron paramagnetic resonance, FSC: forward scatter, SSC: side scatter, PMT: photomultiplier tube, VST: variance stabilizing transformation, DEG: differentially expressed gene, BN-PAGE: blue native polyacrylamide gel electrophoresis, PRDX5: peroxiredoxin 5, GDH: glutamate dehydrogenase, Trx2: thioredoxin 2, COXIV: cytochrome c oxidase subunit IV, PRDX3: peroxiredoxin 3, Gpx4: glutathione peroxidase, TXNRD2: thioredoxin reductase 2, TXNIP: thioredoxin-interacting protein, CAT: catalase, AcylK: acetyl-lysine, IP: immunoprecipitation, DCPIP: 2,6-dichlorophenolindophenol, MDA: malondialdehyde, GO: gene ontology, NES: normalized enrichment score, SDHB: succinate dehydrogenase B, OCR: oxygen consumption rate.
